# Extraction of pore-morphology and capillary pressure curves of porous media from synchrotron-based tomography data

**DOI:** 10.1038/srep10635

**Published:** 2015-06-03

**Authors:** Feifei Yang, Ferdinand F. Hingerl, Xianghui Xiao, Yijin Liu, Ziyu Wu, Sally M. Benson, Michael F. Toney

**Affiliations:** 1 National Synchrotron Radiation Laboratory, University of Science & Technology of China, Hefei, Anhui 230027, China; 2 Department of Energy Resources Engineering, Stanford University, 473 Via Ortega, Stanford, CA 94305-2205, USA; 3 Advanced Photon Source, Argonne National Laboratory, 9700 S Cass Avenue, Argonne, Illinois 60439, USA; 4 Stanford Synchrotron Radiation Lightsource, SLAC National Accelerator Laboratory, Menlo Park, CA 94025, USA; 5 Beijing Synchrotron Radiation Facility, Institute of High Energy Physics, Beijing 100049, China

## Abstract

The elevated level of atmospheric carbon dioxide (CO_2_) has caused serious concern of the progression of global warming. Geological sequestration is considered as one of the most promising techniques for mitigating the damaging effect of global climate change. Investigations over wide range of length-scales are important for systematic evaluation of the underground formations from prospective CO_2_ reservoir. Understanding the relationship between the micro morphology and the observed macro phenomena is even more crucial. Here we show Synchrotron based X-ray micro tomographic study of the morphological buildup of Sandstones. We present a numerical method to extract the pore sizes distribution of the porous structure directly, without approximation or complex calculation. We have also demonstrated its capability in predicting the capillary pressure curve in a mercury intrusion porosimetry (MIP) measurement. The method presented in this work can be directly applied to the morphological studies of heterogeneous systems in various research fields, ranging from Carbon Capture and Storage, and Enhanced Oil Recovery to environmental remediation in the vadose zone.

The elevated level of atmospheric carbon dioxide (CO_2_) has caused serious concern of the progression of global warming, which could result in severe outcomes such as rising of sea levels and climate changes^1^. While great efforts have been invested in the research for new inexpensive, reliable and sustainable energy source^2^, strategies to manage the atmospheric CO_2_ concentration have been proposed^3^. Geological sequestration is considered as one of the most promising techniques for mitigating the damaging effect of global climate change.

It is well known that the geologic carbon storage (GCS) is a scientific subject that needs to be investigated at a wide range of length-scales due to the heterogeneity of the underground geological formations over broad length-scales. A large amount of laboratory experimental studies are performed at (sub-) core scale^4^, providing important understanding of the behavior of the geological samples at this length-scale, such as the multiple phase flow phenomena. However, the origin of the observed phenomena at (sub-) core scale is not very understood, and needs more information at finer length-scales for further interpretation.

Thanks to the advances in the experimental techniques, e.g. synchrotron based micron-scale X-ray tomography, fine 3D morphology of the specimens can be retrieved with good quality at good resolution^5^. The synchrotron based micron-scale X-ray tomography provides insight into the morphological buildup of rocks, and allows the extraction of various pore space properties, such as porosities, capillary pressures and relative permeabilities. This has also led to a tremendous increase in interest in doing pore scale modeling^6^. The existing methods for doing the modeling have, however, various shortcomings.

The numerical approaches can be divided into two broad categories, direct simulation algorithms and pore-network algorithms. Direct simulation algorithms generally solve the governing equations of flow and transport directly on the micro CT image itself. Among the most common and successful methods in this category are particle-based methods, such as the Lattice Boltzmann method^7^,^8^,^9^,^10^,^11^,^12^,^13^. This very powerful method provides access to a large set of pore space properties, is applicable to even highly complex pore geometries and is relatively straightforward to implement^14^, but is computationally rather expensive and parallelized versions running on computer clusters are required for the simulation. In addition, application of constant-pressure and no-slip boundary conditions is rather challenging^15^,^16^,^17^.

Another common method consists of directly solving the Navier-Stokes equation (which reduces to the Stokes equations at slow flow) on a discretized grid^6^,^18^. Manwart *et al.*^19^ showed that Lattice-Boltzmann and Navier-Stokes based simulations produce similar results at similar computation times; however, the Lattice Boltzmann approach requires in their case about 2.5 times more memory. Given the large size of the micro CT data, this can lead to major constraints with respect to practical application of the method.

Further—less common—methods are the Smoothed Particle Hydrodynamics method^20^, Level set method^21^, Volume of fluid method^22^, and Density functional modeling^23^.

For the second category of models, the pore network modeling approaches, first a topologically representative network is extracted from the micro CT image. The network is then used for computation of the relevant displacement and transport equations. Pore-network models require therefore extensive image pre-processing (network generation) to discretize the pore space into simple geometrical objects (typically nodes and bonds). Each extracted geometrical object will then be represented by effective physical parameters and simplified versions of the respective transport equations will be solved. This technique has been applied to a large amount of reservoir rocks and predictions of capillary pressure and relative permeability are possible^24^,^25^,^26^,^27^,^28^,^29^,^30^,^31^,^32^,^33^.

Blunt *et al.*^34^ subdivide network models into several groups. Grain-based approaches^35^ start by scanning for alleged pores based on their distance to the grain center. According to Blunt *et al.*^34^, this approach works particularly well for pore networks extracted from object-based simulation of grain packing and diagenesis, but is less suitable for networks from micro CT images. This shortcoming is particularly pronounced for rocks with complex pore-grain geometries, such as carbonates.

Erosion-dilation is an approach tailored for identifying the skeleton of the pore space, which forms a virtual line connecting the centers of the connected pores and pore throats^36^,^37^. Blunt *et al.*^34^ argue that a major shortcoming of this method is its constrained ability to unambiguously distinguish between pores and pore throats.

The maximal-inscribed-spheres method^38^,^39^ uses the micro CT image and characterizes the pore space by growing spheres, centered in the voids, in its binarized equivalent. Smaller spheres contained in larger ones are subsequently eliminated, which results in a chain of spheres, where larger ones identify the larger pores and smaller ones the pore throats. Sphere radii can then be related to capillary pressures by means of the Laplace-Young equation.

As reviewed above, current pore network models can provide reasonable values for computed multiphase flow properties, but independent verification is still indispensable. Moreover, due to their simplification of the pore space a unique representation of the latter cannot be guaranteed.

In this paper we propose a new approach that can predict capillary pressures without complex simulation, solely using the rock morphology given by synchrotron based micro CT data. Our approach does neither require solving conservation equations, nor does it depend on the extraction of pore-networks or simplification of process-controlling physics. In this paper we present the methodology underlying our new approach and demonstrate its potential at the example of a capillary pressure curve of heterogeneous sandstone. We also expect that the proposed method can be applied directly to the morphological studies of heterogeneous systems in various research fields, ranging from Carbon Capture and Storage, and Enhanced Oil Recovery to environmental remediation in the vadose zone.

## Results and discussion

**Figure 1:**
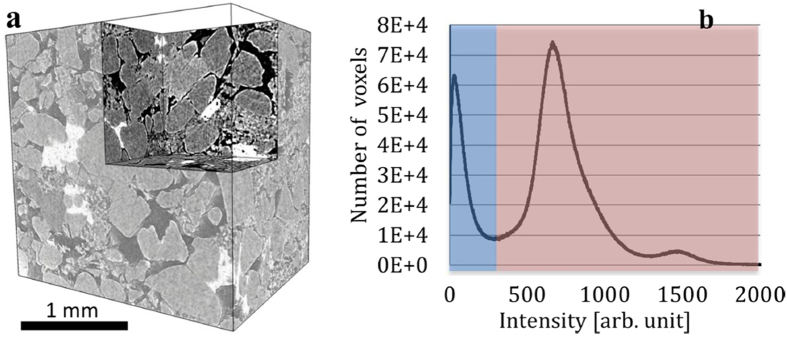
The 3D rendering of a typical reconstructed volume is presented in panel (**a**); the intensity histogram plot of the corresponding 3D volume is presented in panel (**b**), showing well separated peaks for the pore space (blue) and the solid voxels (red). The scale bar in panel (**a**) is 1 mm.

The Heletz structure has been selected as a test site for a prospective CO_2_ reservoir and for the MUSTANG injection experiment based on the analysis of the available geological, geophysical and borehole data from various areas of Israel^40^. In this study, the Sandstone samples were collected from one of the Heletz deep injection wells and screened using medical CT for identifying areas of greatly differing capillarity before small volumes (1 cm in size) of the sample were extracted. The samples of interest were then investigated using synchrotron based micro tomography technique, which provides direct visualization of the three-dimensional (3D) internal morphology at micro-scale (see the 3D rendering shown in Fig. 1a). The details of the experiment are described in the method section.

### The representativeness

**Figure 2:**
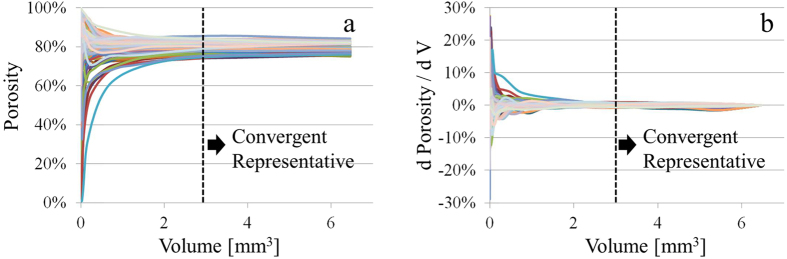
The plots of porosity versus the sample volume for all the 3D matrixes (as indicated by different colors in the plots) investigated in this study. The differential plots are presented in panel (b) with the dash line indicates the convergence and the minimum representative volume at about 3 mm^3^.

The ultimate goal of this study is to understand the relationship between the micro morphology and the phenomena observed at a larger length-scale, the experimental data of the well-known mercury intrusion porosimetry method. We also aim to develop a method capable of predicting the macroscopic behavior (the capillary pressure curve) of the Sandstone samples using the fine morphology retrieved from the synchrotron based micro tomography experiments. In this attempt to correlate the information at very different length scales (the difference at 3 orders of magnitude or larger), it is essential to make sure the representativeness of the relatively small regions investigated, in order to guarantee that the conclusions are meaningful and scalable. In this study, we determine the minimum representative volume by evaluating the porosity as a function of the sample volume^41^. As shown in Fig. 2 (the plots of porosity versus volume for all the 3D matrixes reconstructed in this study) the starting porosity value varies a lot (depending on the position of the starting voxels). However, it is convergent upon growth of the sample volume. The differential plot (Fig. 2b) indicates that the minimum representative volume is at about 3 mm^3^, which is smaller than the size of the 3D matrixes we studied.

### The solid expansion method

The good image contrast achieved is represented by the relatively well separated peaks in the histogram plot (Fig. 1b), which make it straightforward to determine a threshold value to perform segmentation of the data for determining the 3D pore network in the sample. However, the complexity in the morphology of the sample makes it difficult to understand and evaluate the pore structure quantitatively. It has been shown that the porosity is not solely responsible for the single-phase (e.g. permeability) and multi-phase behavior (e.g. capillary pressure curve) of the samples^42^. The effect of the pore structure/morphology, especially of the pore throat structure, is not very well understood and lacks direct quantitative visualization and evaluation. In this study, we present a mathematic method, in which the solid phase is expanded numerically layer by layer, in order to evaluate the pore throat size distribution with statistics.

**Figure 3:**
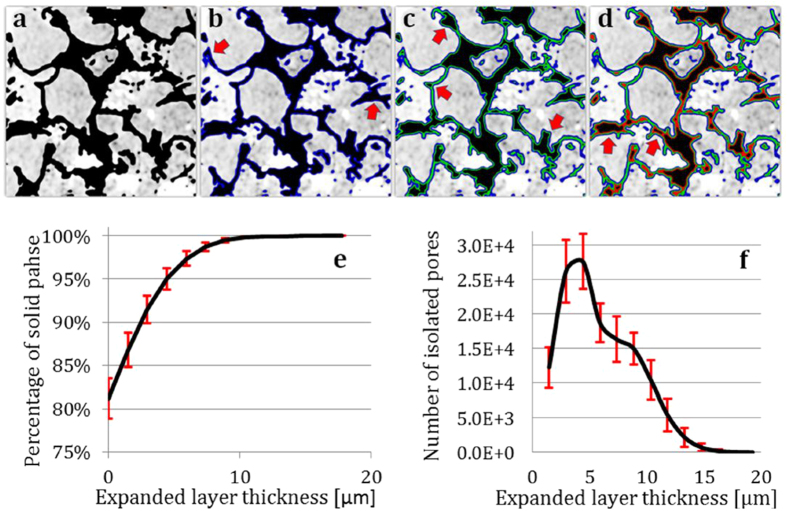
Diagrammatic sketch of the solid (white) expansion method is shown in panels (**a**) to (**d**). Panel (**a**) shows the starting geometry, which is a well-connected pore. Panels (**b**) to (**c**) show the isolated pore(s) is/are formed upon the numerical expansion of the solid phase. The red arrows in panels (**b**) to (**d**) point to the isolated pores formed by closing of the pore throats. Panel (**e**) is the plot of the percentage of the solid volume over the entire 3D space as a function of the solid layer expansion. Panel (**f**) is the plot of the number of isolated pores versus the number of voxel layers expanded numerically. The error bar is the standard deviation of the data from more than 70 of the 3D matrices.

As shown in the schematic drawing in Fig. 3a–d, the well-connected pore (Fig. 3a) is divided into several isolated pieces as the solid phase is expanded numerically. Isolated pores are formed upon the expansion of the solid phase, caused by closing of the pore throat(s) (Fig. 3b–d, as indicated by the red arrows). Eventually, the pores will disappear, because all the void space will be occupied by the (numerically expanded) solid voxels. It is naturally expected that the number of isolated pores will increase then decrease upon numerical expansion of the solid phase.

The above described method is applied to the tomography data on the sandstone samples. As shown in Fig. 3f, the number of isolated pores did increase and then decrease upon numerical expansion of the solid phase. There are two separated peaks in this plot shown in Fig. 3f, which indicate the presence of two types of pore throats in the sample, the primary pore throat (pore throats at fine length scale comparable to the spatial resolution in this study) and the secondary pore throat (at a larger scale). We also show in Fig. 3e the percentage of the solid volume over the entire 3D space as a function of the expanded layer thickness. As more and more space is occupied by the “solid” voxels, this plot goes toward 100% upon numerical expansion.

### Solid expansion and contraction method

The solid expansion method described above provides an intuitive evaluation of the size distribution of the pore throats. However, we realize that the quantitativeness of this method is questionable because the accumulative volume of those surface voxels is considerable comparing to the total volume of the void space within the sample due to the complexity of the pore structure. For predicting the pressure curve quantitatively, we present here an improved approach in which selected numerical solid contraction is performed, in addition to the solid expansion described above, to overcome the problem discussed above.

**Figure 4:**
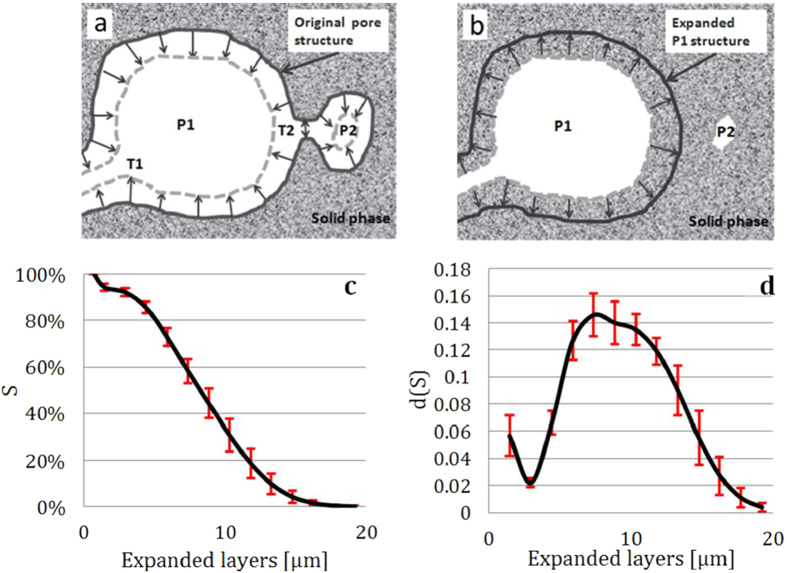
Schematic illustration of the quantitative evaluation of the pore structure using the proposed solid expansion and contraction method is shown in panels (**a**) and (**b**). Panel (**c**) shows the plot of the normalized accessible pore volume (the saturation percentage) versus the pore throat. The differential plot of panel (**c**) is shown in panel (**d**). The error bar is determined by analyzing the standard deviation of the experimental data from the tomographic measurement of different areas of the sample.

As schematically shown in Fig. 4a,b, the solid expansion of voxel layer(s) is/are followed by the same amount (same amount of voxel layer(s)) of solid contraction, but, over the largest connected pore (the main pore). The detailed description of the solid expansion/contraction algorithm can be found in the supplementary information. By doing this, the amount of surface-caused volume loss in our numerical approach is compensated. This procedure is conducted iteratively on the largest connected pore to quantitatively calculate the saturation percentage (the normalized accessible pore volume, S) versus the pore throat radius (r) as shown in Fig. 4c, and the corresponding derivative data shown in Fig. 4d. It is useful to mention that it takes less than a minute to complete this calculation for a volume of 500 × 500 × 500 voxels using a standalone workstation with 64 GB of ram and 12 processors.

### Comparison with the traditional method

**Figure 5:**
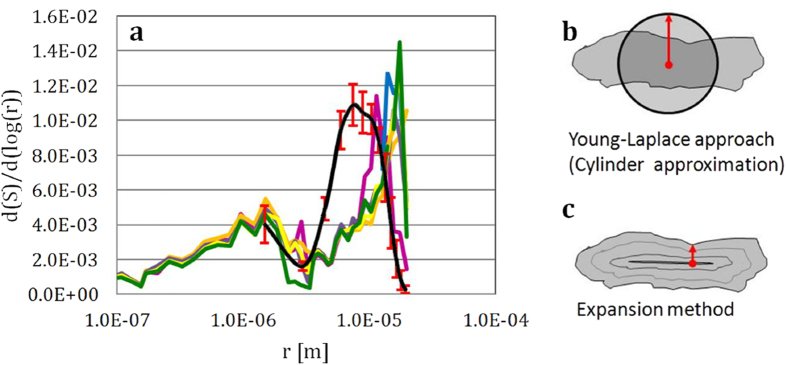
Panel (**a**): The comparison of the capillary pressure curves generated by the solid expansion and contraction method (the black curve with the error bar indicated in red) and by the mercury intrusion experiments (the colored curves). Panels (**b**) and (**c**) illustrate the different methods used to calculate the effective pore throat radius in the Young-Laplace approach and the solid expansion and contraction method respectively.

As described in the section above, the capillary pressure curve can be generated by the calculation following the definition (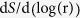 versus 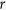, where S is the saturation volume and r is the pore through size), as shown in Fig. 5a. For better evaluation of the proposed method, we compare our result with the experimentally measured capillary pressure curves on a few sandstone cylinders of the same kind, which are studied using the conventional mercury intrusion method^43^ (Fig. 5a).

In a Mercury intrusion experiment the mercury is progressively forced into the porous structure under stringently controlled pressures. The required equilibrated pressure is directly related to the size of the pores. From the pressure and the saturation volume, the pore size distributions (capillary pressure curves) are generated using the Young-Laplace equation, in which the cylinder approximation is included^43^.

In General, the curve generated using the solid expansion and contraction method shows the two peaks of capillary pressure curves (Fig. 5a), which indicates that there are two types of pore throats in the specimens. These two types of pore throats are at different length scales (the primary pore throats at ~1 micron and the secondary pore throats at ~10 microns), which is in good agreement with the capillary pressure curves generated from the mercury intrusion experiments. However, there is a considerable shift (by a factor of ~2) of the second peak representing the secondary pore throats to smaller value.

This is likely caused by the difference in the ways pore throat size is calculated in these two methods. As illustrated in Fig. 5b, the cylinder approximation in the Young-Laplace equation uses the circular cross-section with comparable area to approximate the radius value, which could be significantly off in reality because of the complexity in the pore morphology. On the other hand, the solid expansion and contraction method (proposed in this work) takes the pore structure into consideration and yields the radius value that relates to the narrowest part of the pore throat geometry (Fig. 5c). In other words, the cylinder approximation is likely to overestimate the effective pore throat radius, while the solid expansion and contraction method may be a more reasonable alternative.

**Figure 6:**
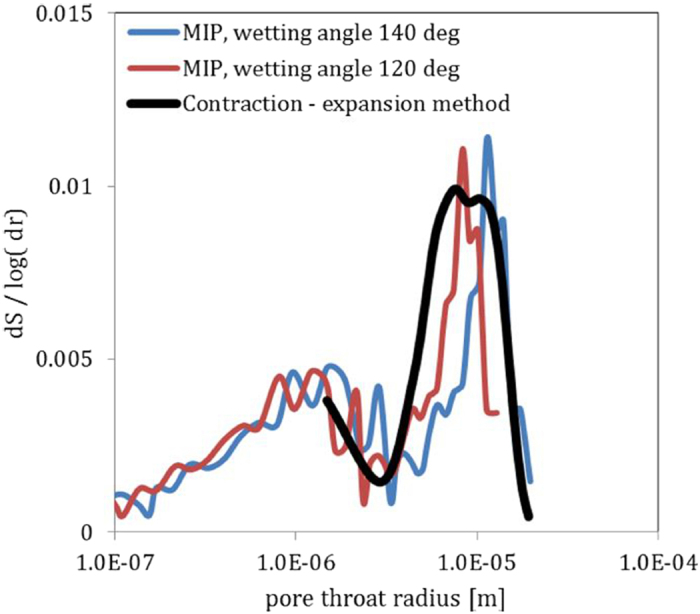
A change of wetting angle translates into a shirt of the computed pore throat radius distribution towards smaller radii. Given in blue is the curve computed form experiment 1 (Fig. 5) using a wetting angle of 140 degree. The red curve uses the same experimental values, but employs a wetting angle of 115 degree in the Laplace-Young equation. The values coming from the solid contraction and expansion method are given in green.

In addition to the cylinder approximation, further simplifying assumptions are made when using the Laplace-Young equation to compute pore throat radii from capillary pressure measurements. First, a surface tension value for mercury of 0.485 Nm^−1^ is generally used. According to Giesche, 2006^43^, this value is widely accepted and not subject to significant variation at 25 degree C. Second, a contact angle of 140 degree is employed. As studies have shown, however, this value can vary by over 10 degrees even for the same material^44^. Figure 6 shows that a wetting angle of 120 degree shifts the dS/log(dr) values (curve exp1 from Fig. 5a) towards smaller pore throat radii, which can explain the difference between the new Solid Expansion & contraction method and the Mercury Intrusion Porosimetry method.

**Figure 7:**
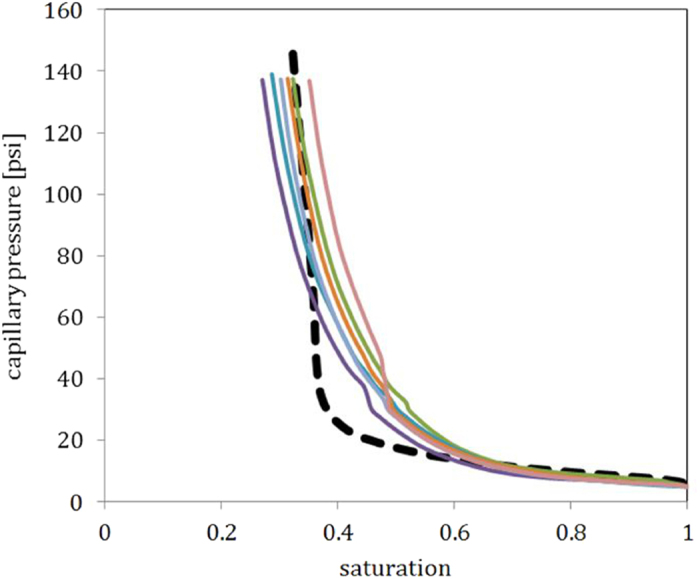
Comparison of the capillary pressure curves as computed by mercury intrusion (colored curves) and solid contraction and expansion method (black curves, the solid and the dotted curves are the measurement over different sub-volumes of the sample).

Figure 7 compares the different capillary pressure curves measured by mercury intrusion (in color) and computed by our new method (shown in black are the curves for our measurements). The curves clearly overlap at high saturations, but diverge between 0.6 and 0.4. MIP measurements indicate a smooth change in capillary pressure (indicative for a more heterogeneous pore throat distribution), while the computed profile is rather flat with a steep increase in capillary pressure at a saturation of 0.37. Both methods seem to converge at a similar value for the irreducible wetting phase saturation. Furthermore the capillary measurements by MIP exhibit a kink at around 30 psi, which results from a manual change in pressure chamber used for the mercury injection.

Another factor possibly contributing to the difference between the two methods lies in the segmentation procedure used for generating a binary image (pore versus rock phase) from the original grey-scale micro CT image. A small shift in the threshold value distinguishing the two phases can lead to considerable over-/underestimations of the size of pore space and therefore contribute to a noticeable error particularly in the case of small pores and pore throats. This uncertainty together with the technical error inherent in the method at the resolution limit of the micro CT imaging method can also cause the difference in the results.

## Summary and conclusion

We developed a (numerical) surface expansion and contraction method to evaluate the pore sizes distribution of a porous structure using synchrotron based micro tomography data and demonstrated its capability in predicting the capillary pressure curve of heterogeneous morphology. The proposed method is simple, intuitionistic, and capable of providing pore throat radius distributions directly without any approximation or complex calculation that required large computation time. Comparison of the proposed method and the conventional experimental method shows reasonably good agreement. The origin of difference in the result is also discussed.

The proposed method provides good understanding of the micro structure and macro behavior. It is capable of predicting the macro behavior of the sample by analyzing the micro structure. The proposed method can be applied directly to the morphological studies of heterogeneous systems in various research fields, including Carbon Capture and Storage, Enhanced Oil Recovery, Environmental Remediation in the vadose zone, Heterologous Catalysis and beyond. It is also important to mention that the numerical method is independent to the imaging platforms. As a result, we expect this method to be extendable and scalable for more complicated heterogeneous system at different length scales by using the imaging results from different instruments as input to our method (especially with the developments of advanced X-ray focusing optics^45^,^46^ that enables the X-ray microscopy at much finer length scales).

## Methods

Synchrotron based micro-tomography experiments were performed at the beamline 2-BM-B of the Advanced Photon Source at the Argonne National Laboratory. In this experiment, monochromatic X-rays with energy of 31 keV were used for imaging. The choice of X-ray energy is made with consideration of the tradeoff between the penetration capability and the image contrast. On one hand, the requirement of the representativeness has set the lower limit of the sample size and, thus, the lower limit of the X-ray energy. On the other hand, X-rays at lower energy tend to provide better absorption contrast. We choose X-rays at 31 keV to ensure good quality (as evidenced by the well separated peaks in the histogram plot, indicating good Signal to Noise ratio) of the imaging data over meaningful sample size. In general, phase contrast, which is a routine imaging mode at synchrotron imaging facilities, can be utilized to further alleviate the contrary requirements to x-ray energy from penetration and contrast considerations. The objective lens employed provides a magnification of 2.5×, resulting in a field of view (FOV) of 3 × 6 mm^2^ and spatial resolution of about 2.96 × 2.96 micron^2^. In a tomography scan, 1501 images were collected over an angular range of 0°–180°, which allows tomography reconstruction (using standard Filtered Back Projection algorithm^47^, the FBP) with good quality. For sub-pixel accuracy in the numerical analysis (to be presented in this paper), we perform bi-cubic interpolation of the data which results in a voxel size of 1.88 × 1.88 × 1.88 micron^3^.

For better statistic, multiple samples were scanned in this study. More than 70 of the 3D matrixes (size at 710 × 710 × 550 voxels; volume at ~6.5 mm^3^) were reconstructed using an in-house developed software package known as TXM-Wizard^48^. The rendering of a typical 3D volume is presented in Fig. 1a. As shown in Fig. 1a, the micro tomography image resolves the fine features in the scanned area and provides good image contrast for further analysis and segmentation of the solid phase versus the void space (the pores), as indicated by the well separated peaks in the intensity histogram plot (Fig. 1b).

## Additional Information

**How to cite this article**: Yang, F. et al. Extraction of pore-morphology and capillary pressure curves of porous media from synchrotron-based tomography data. *Sci. Rep.* **5**, 10635; doi: 10.1038/srep10635 (2015).
